# A Definitive Volume on Temperament

**DOI:** 10.3389/fpsyg.2012.00332

**Published:** 2012-09-18

**Authors:** Michael I. Posner

**Affiliations:** ^1^Department of Psychology, University of OregonEugene, OR, USA

If you wanted to predict whether a 5-year-old child would have a healthy life, earn a large income or be admitted to Harvard, what data would you use? Would it help most to know their genome, have a brain scan or ask the parent to fill out a temperament questionnaire. Based on the new *Handbook of Temperament* the somewhat surprising answer might be that a temperament measure of effortful control might be the best predictor. It would also save you the one to two thousand dollar cost of the other measures.

These days brain measures are popular, but perhaps not at the right level of analysis to understand, measure, predict, and prescribe for the developing child. In the overview chapter, Mary K. Rothbart defines temperament as “constitutionally based individual differences in reactivity and self regulation, influenced over time by genes, maturation, and experience (Rothbart, 2012; page 9).” The editors of the handbook Zentner and Shiner (2012), note that the field of temperament seeks a “comprehensive, concerted effort to identify early appearing, enduring behavioral phenotypes, with a presumed biological basis; to examine their role in psychological development; and to explore their relevance for treatment (page 674).”

Temperament is most often measured by giving caregivers or the person themselves a questionnaire that quantifies the person's behavior on various dimensions of reactivity to stimuli and the ability to regulate or control their behavior. Factor analyses and other statistical methods are used to extract the major components of temperament.

Despite the many complexities raised in this book there does seem to emerge something of a consensus on the dimensions of temperament and on their relation to adult personality. According to Rothbart (2012; page 10) these include activity level, anger/irritation, behavioral inhibition, positive emotionality, effortful control, and perhaps empathy and sensory sensitivity.

The major sections of this volume are labeled Foundation, Basic Temperament Traits, Measures of Temperament, Biological Perspectives, Temperament in Context, Clinical and Applied Perspectives and Integration and Outlook, thus this volume covers pretty much all aspects of the field. The editor have chosen an authoritative group of authors many of them world leaders of the field including in addition to the editors and Dr. Rothbart: Jerome Kagan, Jan Strelau, Marvin Zuckerman, Susan Calkins, Richard Depue, Christina Barr, Hill Goldsmith, Kirby Deater-Deckard, M. Rosario Rueda, Nathan Fox, Jack Bates, and Marinus van IJzendoorn.

In addition to the overview and summary chapters I found particularly helpful the chapter by Zuckerman (2012) on models of adult temperament. His helpful warning to the outside reader that investigators often use different names for the same construct or the same name for different constructs is helpful in comparing the various chapters.

One example of how complex it is to trace the influence of genes on behavior began for me when Zuckerman introduced the Dopamine 4 Receptor Gene (DRD4) in relation to sensation seeking. Sensation seeking is obtained from temperament scales. The DRD4 comes in several versions (called alleles). This makes it possible that the various alleles may somehow be related to individual differences in the degree of sensation seeking. However, after reading several chapters that report findings on the DRD4 I thought of the blind men attempting to describe an elephant. In Chapter 7, the DRD4 is related to anger, in Chapters 6, 17, and 18 to positive affect, in Chapter 13 to activity level, in Chapter 16 to attention deficit hyperactivity disorder, and in Chapter 17 to regulatory processes. In Chapters 6 and 27 one finds that the seven repeat allele may interact with aspects of parenting to influence sensation seeking. After reading Chapter 19, however, which doesn't directly mention this gene, one learns that differences in gene can make the person more susceptible to environmental influence (parenting in this chapter). For example, a person with the seven repeat allele may take more than normal risks if raised in one environment and less than normal if raised in another. Clearly the influence of the gene is much more subtle than causing one to take risks. These complexities support the idea that temperament scales themselves rather than genes may be the right level of analysis for many psychological questions.

This certainly does not mean that genes are not important for many questions. The chapters by Barr (2012) on temperament in animals and by MacDonald (2012) on temperament and evolution, both provide importance perspectives on the precursors of human temperament and genetic influences are clearly a major part of that story. Genes also provide an important links between temperament and the physiological study of the brain and mind, but for many purposes the temperament scales remain the most convenient summary.

Temperament scales as derived from observation or questionnaires seem to provide an important summary of human commonalities and differences. There are many issues involved in their collection and interpretation. This book does an admirable job of making clear both the complexity and the opportunities such scales provide. While the casual reader may be put off by the 750 pages and 32 chapters, I would highly recommend it as a rewarding read for the many of us who want to understand how two children raised by the same parents in one culture can differ so much.
